# Comparison of the safety between propylthiouracil and methimazole with hyperthyroidism in pregnancy: A systematic review and meta-analysis

**DOI:** 10.1371/journal.pone.0286097

**Published:** 2023-05-19

**Authors:** Yue Liu, Qianqian Li, Yang Xu, Yixin Chen, Yanyan Men

**Affiliations:** School of Nursing, Qilu Medical University, Zibo, Shandong Province, China; Sreenidhi Institute of Science and Technology, INDIA

## Abstract

**Objective:**

The purpose of this meta-analysis was to assess the safety of the anti-thyroid drugs (ATDs) propylthiouracil (PTU) and methimazole (MMI) in the treatment of hyperthyroidism during pregnancy.

**Method:**

From inception until June 2, 2022, all available studies were searched in PubMed, Web of Science, Cochrane, EBSCO, Embase, Scopus, and CNKI.

**Result:**

Thirteen articles satisfying the inclusion criteria were examined. Our meta-analysis indicated that pregnant women treated with MMI had a higher risk of congenital anomalies than those treated with PTU (OR 0.80, 95%CI 0.69–0.92, *P* = 0.002, I^2^ = 41.9%). Shifting between MMI and PTU during pregnancy did not reduce the risk of birth defects compared to PTU alone (OR 1.18, CI 1.00 to 1.40, *P* = 0.061, I^2^ = 0.0%). There were no statistically significant differences in hepatotoxicity (OR 1.54, 95%CI 0.77–3.09, *P* = 0.221, I^2^ = 0.0%) or miscarriage (OR 0.89, 95%CI 0.72–1.11, *P* = 0.310, I^2^ = 0.0%) between PTU and MMI exposure.

**Conclusion:**

The study confirmed propylthiouracil is a safer alternative to methimazole for treating hyperthyroidism in pregnant women, and it is appropriate to treat maternal thyroid disease with PTU during the first trimester of pregnancy. However, it is not clear whether switching between propylthiouracil and methimazole is a better option than treating PTU alone during pregnancy. Further studies on this matter may be needed to develop new evidence-based guidelines for the treatment of pregnant women with hyperthyroidism.

## Introduction

Hyperthyroidism is among the most prevalent endocrine disorders in pregnancy, occurring in about 0.2% of pregnant women and being caused primarily by Graves’ disease [1]. It is considered that hyperthyroidism during pregnancy may lead to a variety of fetal and maternal complications including pregnancy loss, preterm delivery, preeclampsia, which requires an important level of awareness for obstetricians [2]. Propylthiouracil and methimazole are commonly prescribed medications to treat hyperthyroidism during pregnancy, with the goal of reducing thyroid hormone synthesis [3]. Both drugs are equally effective in treating maternal hyperthyroidism. Because anti‑thyroid drugs can cross the placenta, the management of hyperthyroidism during pregnancy must be carefully balanced. Traditionally, PTU has been regarded as the best option for treating pregnant women due to its lower rate of placental transfer and less severe PTU-caused teratogenicity than MMI [4]. Congenital abnormalities associated with PTU are felt to be less severe and surgical correctable (i.e. preauricular cysts, urinary tract abnormalities) compared with those of MMI (i.e. aplasia cutis, choanal and esophageal atresia) [2]. However, in the past two decades, an increasing number of reports have raised concerns about hepatotoxicity caused by PTU [5, 6]. Many studies have concluded that women treated with PTU have a higher risk of adverse events than those treated with MMI [7]. The warning may have an impact on the daily clinical practice of endocrinologists in treating hyperthyroidism, calling into question the safe management of pregnant women.

Therefore, it is necessary to update previously published research on the effects of ATDs treatment on the risk of pregnancy outcomes to investigate the contradictory results of using PTU and MMI. In this meta-analysis, we specifically highlight the impact of PTU and MMI conversion use on congenital anomalies, in order to assist clinician provide patients with a better choice of drugs for their individualized treatment.

## Materials and methods

### Systematic search and strategy

The meta-analysis was carried out in accordance with the Preferred Reporting Items for Systematic Reviews and Meta-analysis (PRISMA) guidelines [8]. Two researchers conducted a comprehensive literature review independently. The reviewed databases included PubMed, Embase, Web of Science, Cochrane, EBSCO, Scopus and the Chinese National Knowledge Infrastructure, which were systematically searched to identify relevant articles after inception and before June 2, 2022.

Our search strategy included the following MeSH terms: ("pregnancy" OR "Pregnancies" OR "Gestation") AND ("hyperthyroidism" OR "Hyperthyroid" OR "Hyperthyroids" OR "Primary Hyperthyroidism" OR "Hyperthyroidism, Primary") AND ("Propylthiouracil" OR "6-Propyl-2-Thiouracil" OR "6 Propyl 2 Thiouracil") AND ("Methimazole" OR "1-Methyl-2-mercaptoimidazole" OR "1 Methyl 2 mercaptoimidazole") This research adopts the method of combining free words and theme words. Taking the Pubmed as an example to show the complete literature search process.

### Inclusion and exclusion criteria

The studies chosen for this meta-analysis had to fulfill the following requirements:

1. The women were definitely diagnosed with hyperthyroidism during pregnancy, and the diagnosis adhered to the 2021 European Thyroid Association Guideline [9];
2. Observational studies or randomized controlled studies;
3. A group exposed to propylthiouracil therapy (exposure group); a control group of women exposed to methimazole alone or switched between PTU and MMI therapy (control group), with comparisons between the two groups;
4. At least one of the indicators was included in the outcome measures (congenital anomalies, hepatotoxicity, miscarriage). Congenital anomalies are structural or functional defects that appear during prenatal development. Hepatotoxicity refers to when AST and ALT more than double the upper limit of the reference range [10]. Miscarriage is defined as a spontaneous loss of an intrauterine pregnancy, which occurs before the fetus can survive outside the uterus [11].

Any research that met one of the following exclusion criteria was barred from inclusion in the meta-analysis:

1. Selected treatments were combined with other drugs or supplements.
2. Repeated publications and replicas on various databases.
3. Animal or cell culture studies, reviews, in vitro studies, case reports, meeting abstracts.

### Data extraction and literature quality assessment

Extract data using predefined standardized Excel (Microsoft Corporation, USA) files and resolve differences through discussion. Basic research information (author, title, study type, publication year), experimental design (number of cases, interventions, dosage, duration of exposure), and outcome indicators were extracted from the chosen research papers. The Newcastle-Ottawa Scale (NOS) was used to assess the methodological quality of observational research. NOS consists of three fields and eight items: four items for selection, one item for comparison, and three items for outcomes. The NOS ratings ranged from 0 to 9 stars. All of the research included in the review was evaluated by two authors, and disagreements were resolved through discuss with the research team.

### Statistical analysis

All analyses were performed using STATA, version 12.0 (Stata Corporation, College Station, TX, USA). The statistics of counting data are represented by odds ratio (OR), and the statistics of measuring data are represented by weighted mean difference (WMD). The effect of counting data and measuring data are both expressed as 95% confidence interval (CI). In terms of the heterogeneity test, when the statistics *P* > 0.05, I^2^ < 50%, it can be assumed that the research results have high homogeneity, which means that there is no significant statistical difference in the included data, so the fixed effect model is adopted. When the statistics *P* ≤ 0.05 and I^2^ ≥ 50%, indicating that there are significant statistical differences among the included data and there may be heterogeneity considering the factors that may cause heterogeneity, so the random effect model is used. If a study is significantly different from all other included studies in terms of methods or findings, we conducted a sensitivity analysis to exclude the study from the meta-analysis. To assess publication bias, use forest plot, Egger’s and Begg’s tests, and *P*<0.05 was considered statistically important, unless otherwise defined.

## Results

### Study selection and study characteristics

**Fig 1:**
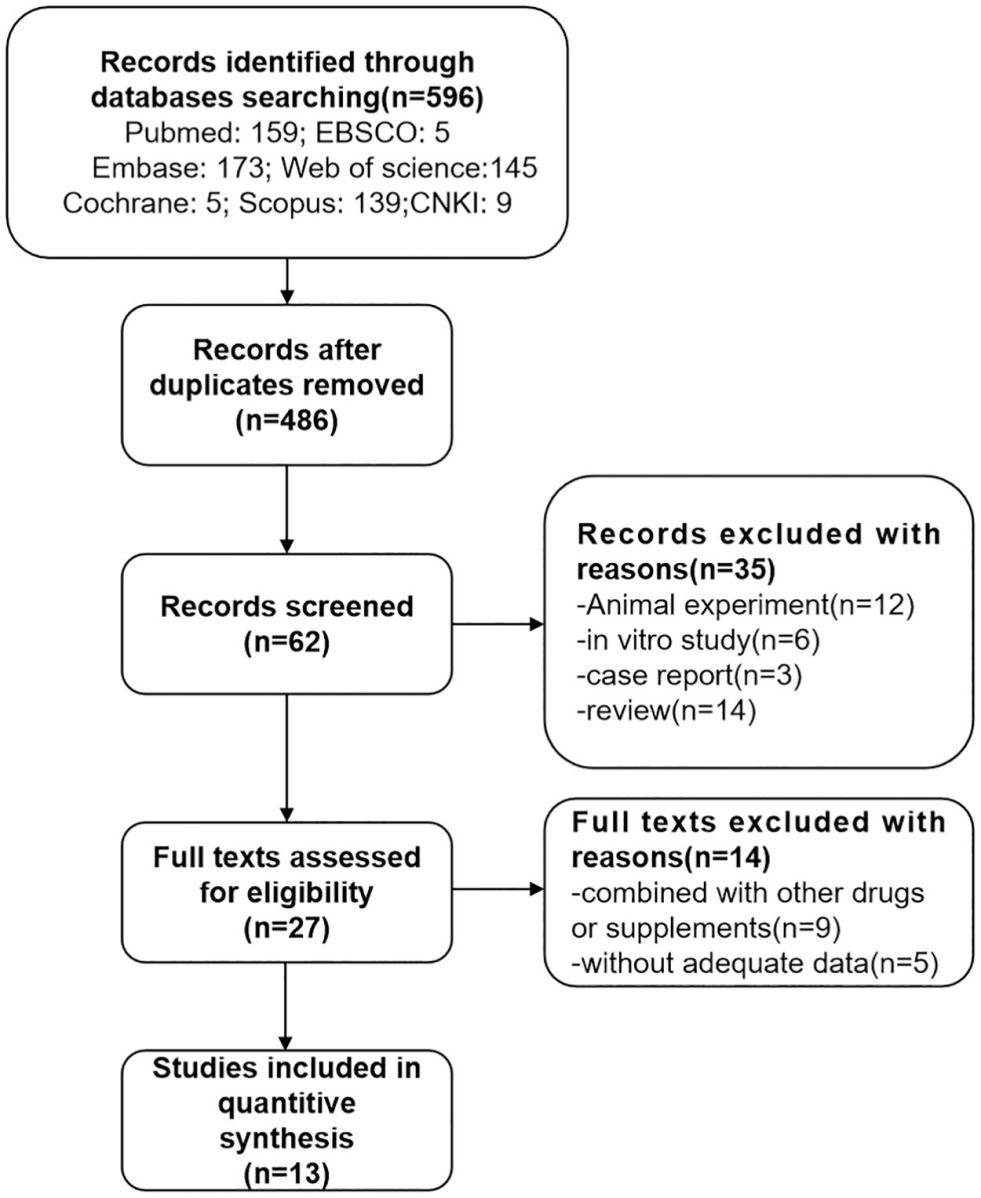
Flow chart of study selection process.

A total of 596 studies were initially identified (PubMed: 159; Embase: 173; Cochrane: 5; EBSCO: 5; Web of science: 106; Scopus: 139; CNKI: 9), After eliminating the repeatedly published articles, 486 articles remained, of which 62 studies were included by screening titles and abstracts. Finally, 13 observational studies were considered to meet the inclusion criteria. Fig 1 describes the selection procedures for the 13 studies using a flow chart. The search strategy is detailed in S1 File, and the PRISMA checklist is shown in the S1 Table.

The summarized characteristics of the studies are provided in Table 1. Ten studies separately reported congenital anomalies of PTU and MMI exposure cohorts [12–21], four studies reported congenital anomalies of PTU and shifting between PTU and MMI exposure cohorts [14, 17–19], and 3 studies reported hepatotoxicity of PTU and MMI exposure cohorts [17, 18, 22], and 4 studies reported miscarriage of PTU and MMI exposure cohorts [21–24]. The results of the NOS used to evaluate the quality of one retrospective cohort study are shown in Table 2.

**Table 1:** Characteristics of included studies.

Study	Year	Study type	Participants			Outcomes
			PTU only	MMI only	PTU and MMI	
Hao	2018	Cohort study	56 women treated with PTU; Dose unknown	22 women treated with MMI; Dose unknown	-	①
Andersen	2017	Cohort study	218 women treated with PTU; Dose unknown; During the first trimester	162 women treated with MMI; Dose unknown; During the first trimester	-	①
Seo	2018	Cohort study	9930 women treated with PTU; Dose unknown; During the first trimester	1120 women treated with MMI; Dose unknown; During the first trimester	1841 women treated with PTU and MMI; Dose unknown; During the first trimester	①
Lo	2015	Cohort study	433 women treated with PTU; Dose unknown; During the first trimester	25 women treated with MMI; Dose unknown; During the first trimester	39 women treated with PTU and MMI; Dose unknown; During the first trimester	① ②
Gianetti	2015	Cohort study	52 women treated with PTU; daily dose ranges from 50 to 200 mg; During the first trimester	124 women treated with MMI; daily dose ranged from 2.5 to 20 mg; During the first trimester	-	③
Andersen	2013	Cohort study	564 women treated with PTU; Dose unknown; During the first trimester	1097 women treated with MMI; Dose unknown; During the first trimester	159 women treated with PTU and MMI; Dose unknown; During the first trimester	①
Zhu	2017	Cohort study	64 women treated with PTU; The initial dose is 300mg/d, gradually reduced to 25~50mg/d; 2 months to 9 years	64 women treated with MMI; The initial dose is 15~30mg/d, gradually reduced to 2.5~5mg/d; 1 months to 9 years	-	② ③
Andersen	2019	Cohort study	889 women treated with PTU; Dose unknown; During the first trimester	1574 women treated with MMI; Dose unknown; During the first trimester	-	①
Korelitz	2013	Cohort study	915 women treated with PTU; Dose unknown; During the first trimester	108 women treated with MMI; Dose unknown; During the first trimester	126 women treated with PTU and MMI; Dose unknown; During the first trimester	① ②
Yoshihara	2012	Cohort study	1399 women treated with PTU; Dose unknown; During the first trimester	1231 women treated with MMI; Dose unknown; During the first trimester	-	① ③
Chen	2011	Cohort study	630 women treated with PTU; Dose unknown; During the first trimester	73 women treated with MMI; Dose unknown; During the first trimester	-	①
Liang	2016	Cohort study	40 women treated with PTU; The initial dose is 30mg/d, gradually reduced to 5~10mg/d; During the first trimester	40 women treated with MMI; The initial dose is 300mg/d, gradually reduced to 50~100mg/d; During the first trimester	-	③
Wing	1994	Cohort study	99 women treated with PTU; The median maximal daily medication dose was 450 mg with a range of 150 to 600mg; Before the end of 15 weeks of gestation	36 women treated with MMI; Dose unknown; the median maximal daily dose was 40 mg (range 10 to 60 mg). Before the end of 15 weeks of gestation	-	① ③

Note:

^①^congenital anomalies

^②^hepatotoxicity

^③^miscarriage

**Table 2:** Assessment of methodological quality by NOS.

Study	Selection				Comparability	Outcome			Total
	Exposed cohort representativeness	Non exposed cohort selection	Ascertainment of exposure	Outcome not present at start of study	Comparability of cohorts	Assessment of outcome	Follow-up long enough	Adequacy of follow up	
Hao2018	▲	▲		▲	▲	▲	▲	▲	7
Andersen2017	▲	▲	▲	▲	▲▲	▲	▲	▲	9
Seo2018	▲	▲	▲	▲	▲▲	▲	▲	▲	9
Lo2015	▲	▲	▲	▲	▲▲	▲	▲	▲	9
Gianetti2015	▲	▲		▲		▲	▲	▲	6
Andersen2013	▲	▲	▲	▲	▲▲	▲	▲	▲	9
Zhu2017	▲	▲		▲	▲		▲	▲	6
Andersen2019	▲	▲	▲	▲	▲▲	▲	▲	▲	9
Korelitz2013	▲	▲	▲	▲	▲	▲	▲	▲	8
Yoshihara2012	▲	▲	▲	▲	▲	▲	▲	▲	8
Chen2011	▲	▲	▲	▲	▲	▲	▲	▲	8
Liang2017	▲	▲	▲	▲	▲	▲		▲	7
Wing1994	▲	▲	▲	▲	▲	▲	▲	▲	8

### Meta analysis results

Based on 13 cohort studies, the present findings provide strong evidence for the comparison of safety between PTU and MMI treatment.

#### Congenital anomalies

**Fig 2:**
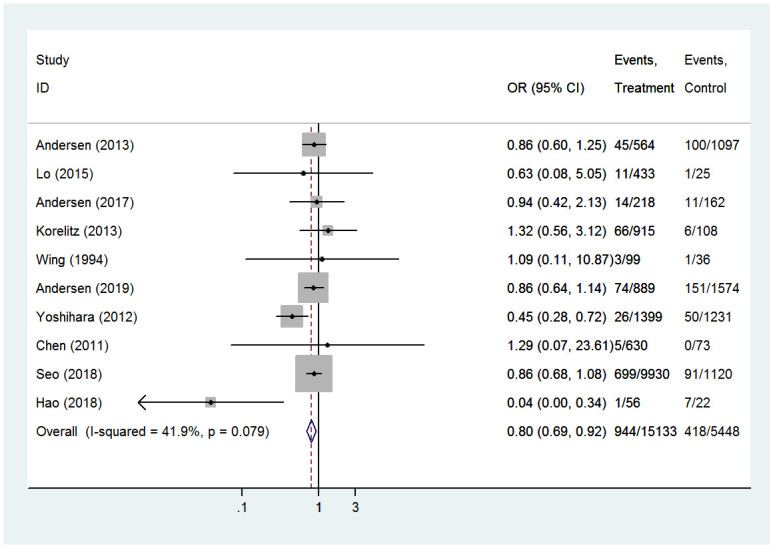
Forest plots of showing the effects of PTU vs MMI on congenital anomalies.

In the present study, the effects of PTU were compared with MMI on congenital anomalies in a total of 20581 infants from 10 studies. Compared with women treated with propylthiouracil, women exposed to methimazole had a higher risk of congenital anomalies (OR 0.80, 95%CI 0.69–0.92, *P* = 0.002) (Fig 2). The fixed effect model was chosen because heterogeneity analysis revealed that the included studies were homogeneous (I^2^ = 41.9%, *P* = 0.079). After removing each individual study, the combined OR of the remaining 9 studies is within the range of the overall effect, which indicates that the results of the meta-analysis were less sensitive and more stable.

**Fig 3:**
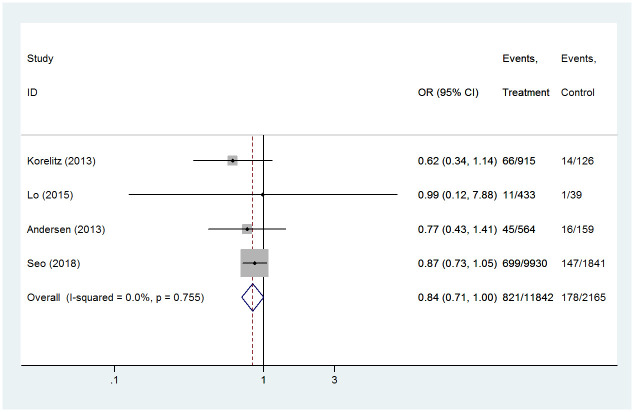
Forest plots of showing the effects of PTU vs shift between PTU and MMI on congenital anomalies.

Since the four studies we included reported on the effect of shifting between MMI and PTU on congenital malformations, we also evaluated this. According to the findings, there was no significant difference in the incidence of congenital abnormality risk among those who switched between MMI and PTU compared with those who received PTU alone (OR 1.18, 95%CI 1.00–1.40, *P* = 0.061) (Fig 3). The heterogeneity test showed that Chi^2^ = 1.19, *P* = 0.775, I^2^ = 0%, indicating that there was no significant heterogeneity among the included research. Sensitivity analysis showed that the combined OR values of the studies remained stable.

#### Hepatotoxicity

**Fig 4:**
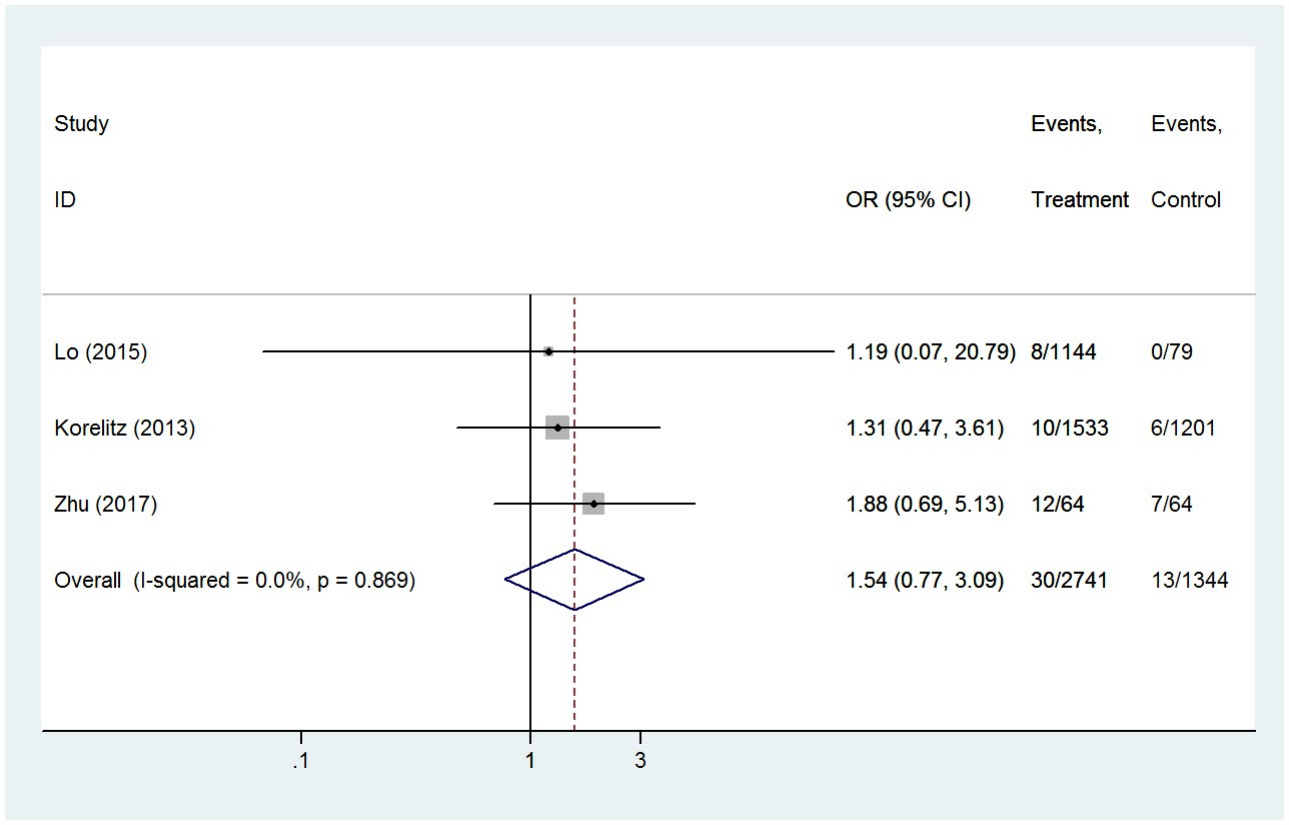
Forest plots of showing the effects of PTU vs MMI on hepatotoxicity.

In the present study, the effects of PTU were compared with MMI on hepatotoxicity with a total of 4085 participants in 3 studies. We assessed differences of hepatotoxicity between PTU treatment group and MMI treatment group, and no significant difference was found (OR 1.54, 95%CI 0.77–3.09, *P* = 0.221) (Fig 4). The heterogeneity between studies was quantified with I^2^ values, and result indicated there was no statistical heterogeneity among studies (I^2^ = 0.0%, *P* = 0.869), so the fixed effect model was chosen.

#### Miscarriage

**Fig 5:**
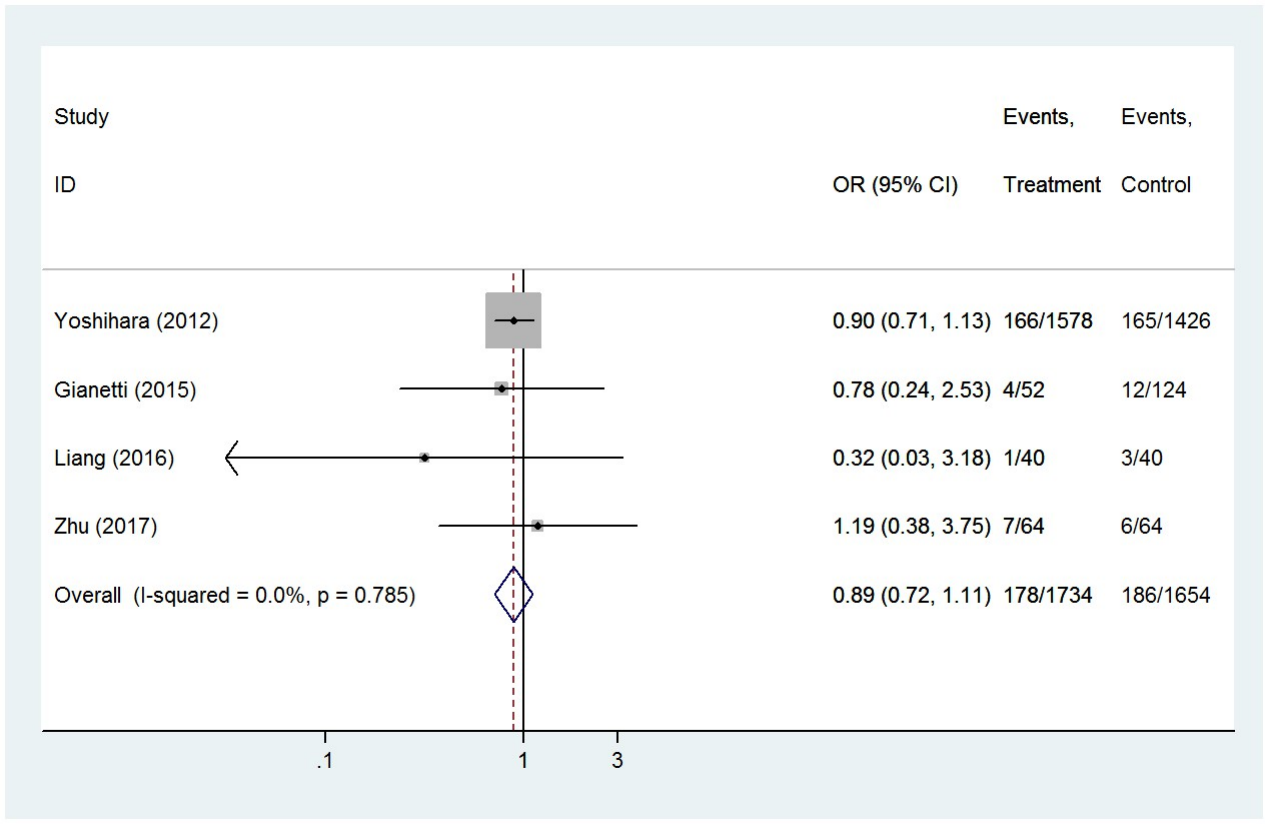
Forest plots of showing the effects of PTU vs MMI on miscarriage.

Four studies reported the findings of miscarriage in pregnancy. There were 178 miscarriages among the 1,734 women who received PTU alone and 186 miscarriages among the 1,654 women who received MMI alone. In pregnant women with hyperthyroidism, there was no significant difference in miscarriage rates between the exposed group (PTU) and the control group (MMI) (OR 0.89, 95%CI 0.72–1.11, *P* = 0.310) (Fig 5). The fixed-effect model was used because there was no statistical heterogeneity among the studies (I^2^ = 0.0%, P = 0.785).

### Publication bias

**Fig 6:**
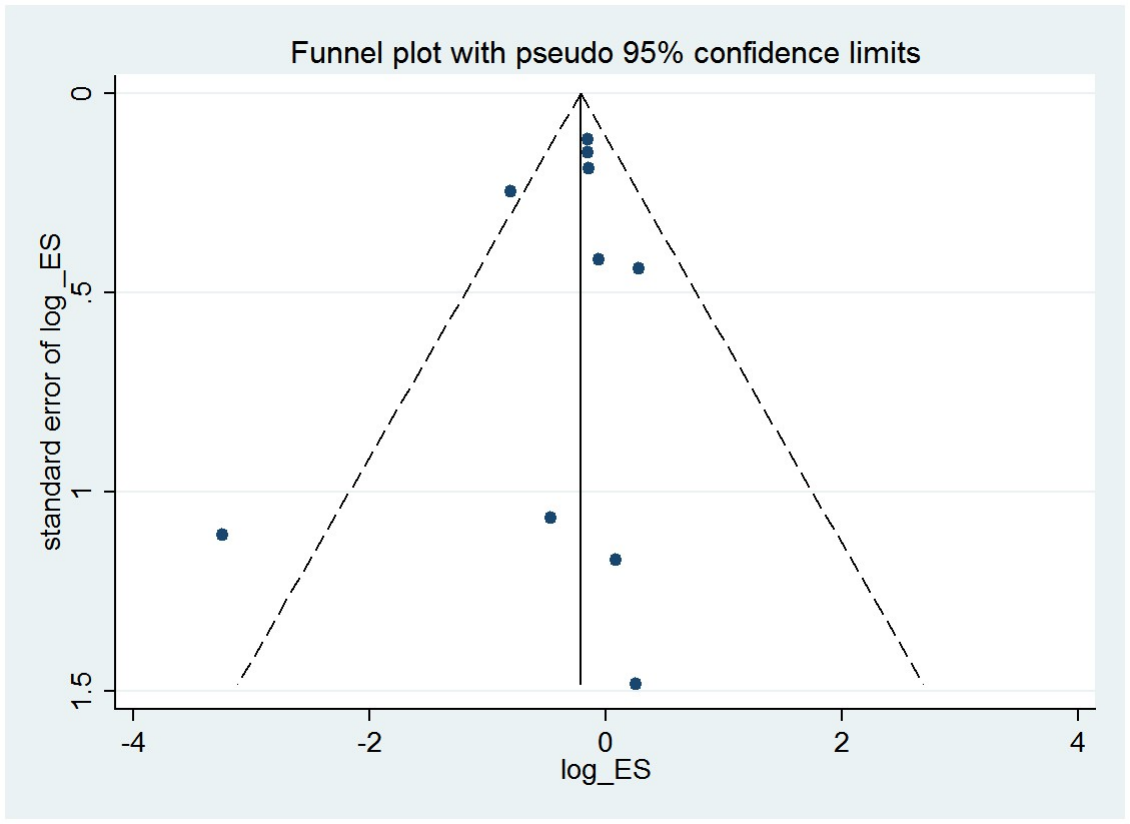
Funnel plot of congenital anomalies of PTU vs MMI.

Fig 6 depicts the funnel plot congenital abnormality risk for PTU and MMI. No obvious asymmetry was found by visual inspection of the funnel plot. The tests of Begg’s and Egger’s also proved that there was no published evidence of bias in the studies of congenital abnormalities risk between PTU and MMI. (*P* = 0.734 and *P* = 0.466, respectively).

## Discussion

PTU and MMI are considered to be the preferred drugs in the treatment of hyperthyroidism in pregnancy [25]. They are also thought to be equally effective in the treatment of combined hyperthyroidism in pregnancy. Propylthiouracil inhibits iodination of tyrosine in the thyroid gland, whereas methimazole prevents oxidation and tyrosine coupling of iodide absorbed into the thyroid gland, which in turn inhibits thyroxine synthesis [26, 27]. The goals of them are to maintain the maternal serum concentration of free thyroxine at the upper limit of the normal range [28]. However, propylthiouracil and methimazole may cross the placenta with similar pharmacokinetics, resulting in adverse pregnancy outcomes [29]. After exposure in pregnancy, MMI could cause a special pattern of teratogenic effects, while rare but severe hepatotoxic sequelae may appear after PTU treatment [30]. Based on this concern, we conducted a meta-analysis of published studies to review the relationship between anti-thyroid drugs and the risk of various adverse outcomes during pregnancy. To summarize, our research showed that the risk of birth defects is higher with MMI treatment than with PTU treatment, and switching ATDs between PTU and MMI did not appear to lower the risk when compared to treated PTU alone. No significant differences were shown in the risk of hepatotoxicity and miscarriage in the MMI group versus the PTU group.

To begin with, our meta-analysis revealed a significant risk of congenital anomalies following exposure to MMI than exposure to PTU. This observation is consistent with the meta-analysis of Song [31]. Morales also proved that although it is still elevated, compared with MMI, PTU has the smallest risk of congenital anomalies, which may be similar to untreated hyperthyroidism [32]. It may be due to the placenta being more permeable to MMI than PTU, and the fetus is more sensitive to the pharmacodynamic effects of MMI [33]. In addition, some studies have specifically reported the types of malformations, exposed to PTU was consistent with urinary tract and head and neck anomalies while the exposed to MMI was associated with classic MMI embryopathy such as choanal and esophageal atresia [14]. The possible cause is unclear and it could be related to the drugs’ mechanism or the drug effects of immunosuppressive [5]. However, the research included in our study did not specifically classify the deformity categories, it could be studied and used as the next step.

Furthermore, we discovered that the recommended approach [34, 35] of switching between MMI and PTU did not decrease the risk of congenital anomalies when compared to PTU alone. The study of Li [36] reached a similar conclusion, they also indicated the risk of birth defects was not associated with use of switching between MMI and PTU. However, some studies have suggested that proper conversion can reduce malformation [37], which may be due to maternal treatment with appropriately timing and have not reached the teratogenic sensitive period. The critical time interval of drug exposure for fetal malformation is between 5–6 weeks and 10 weeks [38]. Actually, the effects of switching can be difficult to explain with these data, because the order and timing of switching was not always specified in our studies. More accurate exposure time is required for further research to clarify the risks associated with switching ATDs.

Several reports provided evidence of severe liver failure related to PTU, which led to a reduction in guidance for PTU use, and suggest that pregnant women should be transferred from PTU to MMI after the first trimester of pregnancy [39]. But in our study, we found that PTU did not increase the hepatotoxicity risk compared with MMI in both the crude and adjusted analyses based on three studies. One of the reasons may be that liver toxicity is related to the patient’s inhibitory media, and both MMI and PTU have the risk of mild liver dysfunction [40]. Accordingly, it is of great value to detect the liver function of women with hyperthyroidism taking anti-thyroid drugs regularly. When transaminase or bilirubin is significantly increased, the drugs should be stopped in time and take effective measures. Another reason could be that, while PTU is related to a severe liver illness’s risk, which could lead to liver failure requiring liver transplants or death, severe liver disease is uncommon [41]. According to research, there is a dose-dependent relationship between ATDs use and the liver dysfunction [42, 43]. Therefore, the dosage of ATDs should be tailored and gradually reduced during pregnancy based on frequent assessments of maternal thyroid function in order to reduce the risk of liver injury.

We found no associations between PTU vs. MMI treatment and miscarriage during pregnancy, indicating that both drugs are equally safe in terms of the risk of miscarriage. There is well-established evidence that women with hyperthyroidism during pregnancy should be treated, as studies have shown that untreated hyperthyroidism may increase the risk of miscarriage [44]. Additionally, the risk of miscarriage has an association with the serum TSH level [45], suggesting that medical personnel should regularly monitor TSH levels in pregnant women to reduce the incidence of adverse pregnancy outcomes. It may be necessary to further study this issue to reveal whether ATDs treatment can improve other pregnancy outcomes of hyperthyroidism women [46].

Our research provides an important population-based assessment of ATDs which shows that it is safer to use PTU during pregnancy than MMI with hyperthyroidism. Furthermore, there is no evidence that switching between PTU and MMI is greater than using PTU alone. We believe our research provides potentially helpful information to physicians prescribing PTU as the first-line treatment for pregnancy, and further studies are required to address whether to switch between PTU and MMI in pregnancy. There are several limitations to our study as well. First, the effects of MMI and PTU on pregnancy outcome change with doses, but the doses of MMI and PTU were not completely uniform in all the studies we included. Secondly, several studies used fetal demise instead of miscarriage as pregnancy outcome, which were excluded due to the failure to extract data.

## Conclusions

Our research provides important population-based estimates of ATDs use that it is safer to use PTU during pregnancy than MMI with hyperthyroidism. Furthermore, there is no evidence that switching between PTU and MMI is greater than using PTU alone. We believe our research provides potentially helpful information to physicians prescribing PTU as the first-line treatment for pregnancy, and further studies are required to address whether to switch between PTU and MMI in pregnancy.

## Supporting information

S1 File Search strategy. (DOCX)

S1 Table PRISMA checklist. (DOCX)

## Abbreviations

ATDs: anti-thyroid drugs
MMI: methimazole
PTU: propylthiouracil
