# Supplementary material for: Comparison of the safety between propylthiouracil and methimazole with hyperthyroidism in pregnancy: A systematic review and meta-analysis
Source: PLoS One. 2023 May 19;18(5):e0286097. doi: 10.1371/journal.pone.0286097 (PMC10198569; doi:10.1371/journal.pone.0286097)
Supplement: S1 File — (DOCX) [file pone.0286097.s001.docx]

Supplementary table1. Search strategy

| Database | Step | Search term | Result(N) |
| --- | --- | --- | --- |
| PubMed | #1 | "pregnancy"[All Fields] OR "Pregnancies"[All Fields] OR "Gestation"[All Fields] | 1072118 |
|  | #2 | "hyperthyroidism"[All Fields] OR "Hyperthyroid"[All Fields] OR "Hyperthyroids"[All Fields] OR "Primary Hyperthyroidism"[All Fields] OR "hyperthyroidism primary"[All Fields] | 37500 |
|  | #3 | "Propylthiouracil"[All Fields] OR "6 propyl 2 thiouracil"[All Fields] OR "6 propyl 2 thiouracil"[All Fields] | 5759 |
|  | #4 | "Methimazole"[All Fields] OR "1 methyl 2 mercaptoimidazole"[All Fields] OR "1 methyl 2 mercaptoimidazole"[All Fields] | 4799 |
|  | #5 | #1 AND #2AND #3 AND #4 | **159** |
| Embase | #1 | "pregnancy" OR "Pregnancies" OR "Gestation" | 1124041 |
|  | #2 | "hyperthyroidism" OR "Hyperthyroid" OR "Hyperthyroids" OR "Primary Hyperthyroidism" OR "Hyperthyroidism, Primary" | 4260 |
|  | #3 | "Propylthiouracil" OR "6-Propyl-2-Thiouracil" OR "6 Propyl 2 Thiouracil" | 808 |
|  | #4 | "Methimazole" OR "1-Methyl-2-mercaptoimidazole" OR "1 Methyl 2 mercaptoimidazole" | **173** |
| Cochrane | #1 | "pregnancy" OR "Pregnancies" OR "Gestation" | 75078 |
|  | #2 | "hyperthyroidism" OR "Hyperthyroid" OR "Hyperthyroids" OR "Primary Hyperthyroidism" OR "Hyperthyroidism, Primary" | 1383 |
|  | #3 | "Propylthiouracil" OR "6-Propyl-2-Thiouracil" OR "6 Propyl 2 Thiouracil" | 140 |
|  | #4 | "Methimazole" OR "1-Methyl-2-mercaptoimidazole" OR "1 Methyl 2 mercaptoimidazole" | 264 |
|  | #5 | #1 AND #2AND #3 AND #4 | **5** |
| EBSCO | #1 | "pregnancy" OR "Pregnancies" OR "Gestation" | 691376 |
|  | #2 | "hyperthyroidism" OR "Hyperthyroid" OR "Hyperthyroids" OR "Primary Hyperthyroidism" OR "Hyperthyroidism, Primary" | 2337 |
|  | #3 | "Propylthiouracil" OR "6-Propyl-2-Thiouracil" OR "6 Propyl 2 Thiouracil" | 325 |
|  | #4 | "Methimazole" OR "1-Methyl-2-mercaptoimidazole" OR "1 Methyl 2 mercaptoimidazole" | 342 |
|  | #5 | #1 AND #2AND #3 AND #4 | **5** |
| Web of science | #1 | "pregnancy" OR "Pregnancies" OR "Gestation" | 544904 |
|  | #2 | "hyperthyroidism" OR "Hyperthyroid" OR "Hyperthyroids" OR "Primary Hyperthyroidism" OR "Hyperthyroidism, Primary" | 22335 |
|  | #3 | "Propylthiouracil" OR "6-Propyl-2-Thiouracil" OR "6 Propyl 2 Thiouracil" | 1507 |
|  | #4 | "Methimazole" OR "1-Methyl-2-mercaptoimidazole" OR "1 Methyl 2 mercaptoimidazole" | 1566 |
|  | #5 | #1 AND #2AND #3 AND #4 | **106** |
| Scopus | #1 | "pregnancy" OR "Pregnancies" OR "Gestation" | 1742763 |
|  | #2 | "hyperthyroidism" OR "Hyperthyroid" OR "Hyperthyroids" OR "Primary Hyperthyroidism" OR "Hyperthyroidism, Primary" | 90937 |
|  | #3 | "Propylthiouracil" OR "6-Propyl-2-Thiouracil" OR "6 Propyl 2 Thiouracil" | 21292 |
|  | #4 | "Methimazole" OR "1-Methyl-2-mercaptoimidazole" OR "1 Methyl 2 mercaptoimidazole" | 4899 |
|  | #5 | #1 AND #2AND #3 AND #4 | **139** |
| CNKI | #1 | pregnancy with hyperthyroidism | 347 |
|  | #2 | propylthiouracil | 1060 |
|  | #3 | Methimazole | 493 |
|  | #4 | #1 AND #2AND #3 | **9** |
